# Genetic monitoring of an endangered arable weed reveals local maintenance of genetic variation in times of land use and climate change

**DOI:** 10.1038/s41598-026-38363-4

**Published:** 2026-02-04

**Authors:** Ellen Gradl, Yoshiko Shimono, Daniela M. Listl, Lena Winkler, Christoph Reisch

**Affiliations:** 1 Institute of Plant Sciences, University of Regensburg, Universitätsstraße 31, 93053 Regensburg, Germany; 2 Graduate School of Agriculture, Weed Science Lab, Kyoto University, Kitashirakawa Oiwake-cho, Sakyo-ku, Kyoto, 606-8502 Japan

**Keywords:** Sherardia arvensis, Rare species, MIG-seq, Conservation genetics, Next-generation-sequencing, Agrobiodiversity, Conservation biology, Ecological genetics, Molecular ecology, Population dynamics

## Abstract

Genetic monitoring is a powerful tool to reveal human impact on genetic diversity and genetic differentiation in times of land use and climate change. Unfortunately, it is not yet frequently applied in wild plant nature conservation at present. Consequently, we conducted a genetic monitoring study of the rare and endangered arable weed *Sherardia arvensis*, which continuously decreased in the recent decades due to environmental changes. Therefore, detecting a possible shift in genetic diversity and differentiation is highly relevant for conservation. We applied multiplexed ISSR genotyping by sequencing (MIG-seq) to compare genetic variation within and among eight populations, as well as effective population size of the species using samples collected at the same sites in 2007 and 2020. We obtained 371 SNPs from 160 analysed individuals. In contrast to our expectations, we observed nearly similar levels of genetic diversity and differentiation within and among populations in 2007 and 2020, although 25% of the investigated populations went extinct in the study period. Effective population sizes showed some differences between the study years, depending on the analysis. The observed maintenance of genetic diversity and differentiation patterns may most likely be explained by the longevity of seeds in the soil, which is generally high in many arable weeds. In the case of *S. arvensis* seeds may persist in the soil for up to ten years. This allows the regeneration of populations and contributes to the maintenance of genetic variation. A potential impact of population loss and decrease of effective population size on genetic diversity and differentiation may therefore be delayed, comparable to the already described phenomenon of an extinction debt in other species. Our study clearly underlines, that long-term genetic monitoring over long time periods is needed to reveal potential changes of genetic diversity and differentiation in the Anthropocene.

**Supplementary Information:**

The online version contains supplementary material available at 10.1038/s41598-026-38363-4.

## Introduction

In the Anthropocene global biodiversity declined drastically due to human impact^1^. Indeed, a dramatic loss of diversity at the ecosystem, species and genetic level in recent decades has been observed and reported in a multitude of different scientific studies^2–5^. Among many others, habitat destruction, land use changes and climate change are the most important reasons for the loss of biological diversity^1^. These processes can cause an extinction vortex^6^, which strongly contributes to species loss. In this case, the progressively decreasing size of a species’ population, first leads to a loss of genetic diversity and increased genetic differentiation, followed by decreasing individual fitness and finally to population extinction. When this process continues, remnant populations become increasingly isolated and suffer from genetic drift and inbreeding, which in turn further increases the likelihood of extinction events^7^. Once started on this downwards spiral, possibilities to reverse the trend are limited. Consequently, for many declining and strongly endangered species, decreasing levels of genetic diversity within populations and increasing levels of differentiation between populations can be postulated nowadays. However genetic diversity is widely recognized as the basis for adaptation and is of high conservation concern. Next to classical genetic diversity and differentiation measures based on genetic markers like expected and observed heterozygosity, the effective population size has been identified as an important parameter by the Convention on Biological Diversity (CBD) in 2022^14–16^. It is particularly useful as a metric to monitor within population genetic diversity as it shows effects of genetic drift, inbreeding and adaptive potential^16,17^.

In this context monitoring, “the systemic and focused observation and measurement of present changes in biodiversity”^8^ is an impressive and effective approach to uncover human impact on the development of biodiversity at all levels^9–11^. At the ecosystem and the species level, monitoring is a well-established and regularly used nature conservation tool. Based upon historical and present maps or aerial photos, the loss and gain of specific ecosystems over time can be analysed^12^. Similarly, historical and present species lists, or vegetation surveys can be used to analyse the occurrence and distribution of species over time at the local, regional or even global level^2^. Genetic diversity and differentiation as an essential part of biodiversity has, however, been largely neglected in biodiversity monitoring up till now^13^.

So far, the genetic monitoring of plant species has mostly focused on crop species^18–20^ or on the effects of ex situ conservation and reintroduction on genetic variation^21–25^. However, genetic monitoring studies, comparing populations of rare and endangered plant species at different times in the field to uncover the temporal development of genetic diversity and differentiation have been conducted less frequently^26^. This can be traced back to several reasons. First, molecular and standardized methods to analyse genetic diversity and differentiation at a broader scale have only become available by and by in recent decades. Second, high quality DNA material, which can be used for genetic monitoring, is often not available. On the one hand, DNA from herbarium specimen is often degraded, in particular when vouchers are old, and cannot easily be used for genetic analyses. Moreover, the number of replications is often too limited for monitoring genetic diversity and differentiation at the population level. On the other hand, collections of DNA solutions are rare and often not old or large enough to study changes in genetic diversity and differentiation over time. Consequently, genetic monitoring studies of rare and endangered plant species are urgently required in conservation^27–29^ and highly significant to analyse changes of genetic diversity and differentiation in a conservation context.

Arable weeds, plant species growing in cultivated fields alongside the crop species, belong to the most endangered plant species in Central Europe^30–34^and are, therefore, highly interesting for genetic monitoring studies. In the modern agricultural landscape, arable weeds strongly declined especially due to land use intensification and other changes^35,36^. More efficient seed cleaning, increased application of fertilizers and herbicides, deeper depth of tillage and the loss of field margin areas due to increased arable field size during land consolidation processes caused an exhaustive decline of arable weed populations all over Central Europe^31,32^.

Our study species, the blue field madder (*Sherardia arvensis*), is an originally quite common arable weed. However, due to agricultural intensification in recent decades the species has become rare and is now included in the German and Bavarian Red List^33,34^. The genetic diversity and differentiation of *S. arvensis* has already been analysed in a previous study using AFLP markers, which revealed a stronger isolation by distance, i.e. higher genetic differentiation with increasing geographical distance, of these arable weed populations in Central Europe compared to the Mediterranean, where many weeds originate from^35^.

For the study presented here, we resampled plant material of *S. arvensis* in 2020 at the study sites in arable field margins, where plant material had already been collected in 2007 for the study of Listl and Reisch^35^. We then applied multiplexed ISSR (inter simple sequence repeats) genotyping-by-sequencing (MIG-seq) to analyse samples from both years and checked for potential changes of genetic diversity and differentiation between 2007 and 2020. Considering the above-mentioned loss of individuals and populations in recent decades, we focused on the following questions: (i) has there been a significant loss of genetic diversity within populations since 2007? (ii) Has the effective population size (N_e_) changed between 2007 and 2020? And finally, (iii) has the level of genetic differentiation among populations increased within this 13-year period?

## Materials and methods

### Study species

Our study species *Sherardia arvensis* L., commonly known as blue field madder, is an annual herbaceous plant species of the Rubiaceae family. The pink to purplish flowers bloom from June to October^40^, while pollination frequently occurs by selfing^41^. *S. arvensis* seeds survive in the soil seed bank up to 10 years^42^.

The species is often considered as an archaeophyte, which was introduced to Central Europe during the Roman Empire at the latest^43^. Due to human agriculture, it is distributed globally. In temperate continental climates it originally occurs in arable fields^39^. However, because of land use changes and agricultural intensification many populations disappeared in the last decades, or they are now restricted to arable field margins. The species is, therefore, included as “near threatened” in the German and Bavarian Red List^37,38^. The collection of the plant material, therefore, did not require permission from local authorities. However, we adhered to relevant institutional and national guidelines to ensure the least possible harm to the studied plant populations.

### Study sites and sampling procedure

**Fig. 1:**
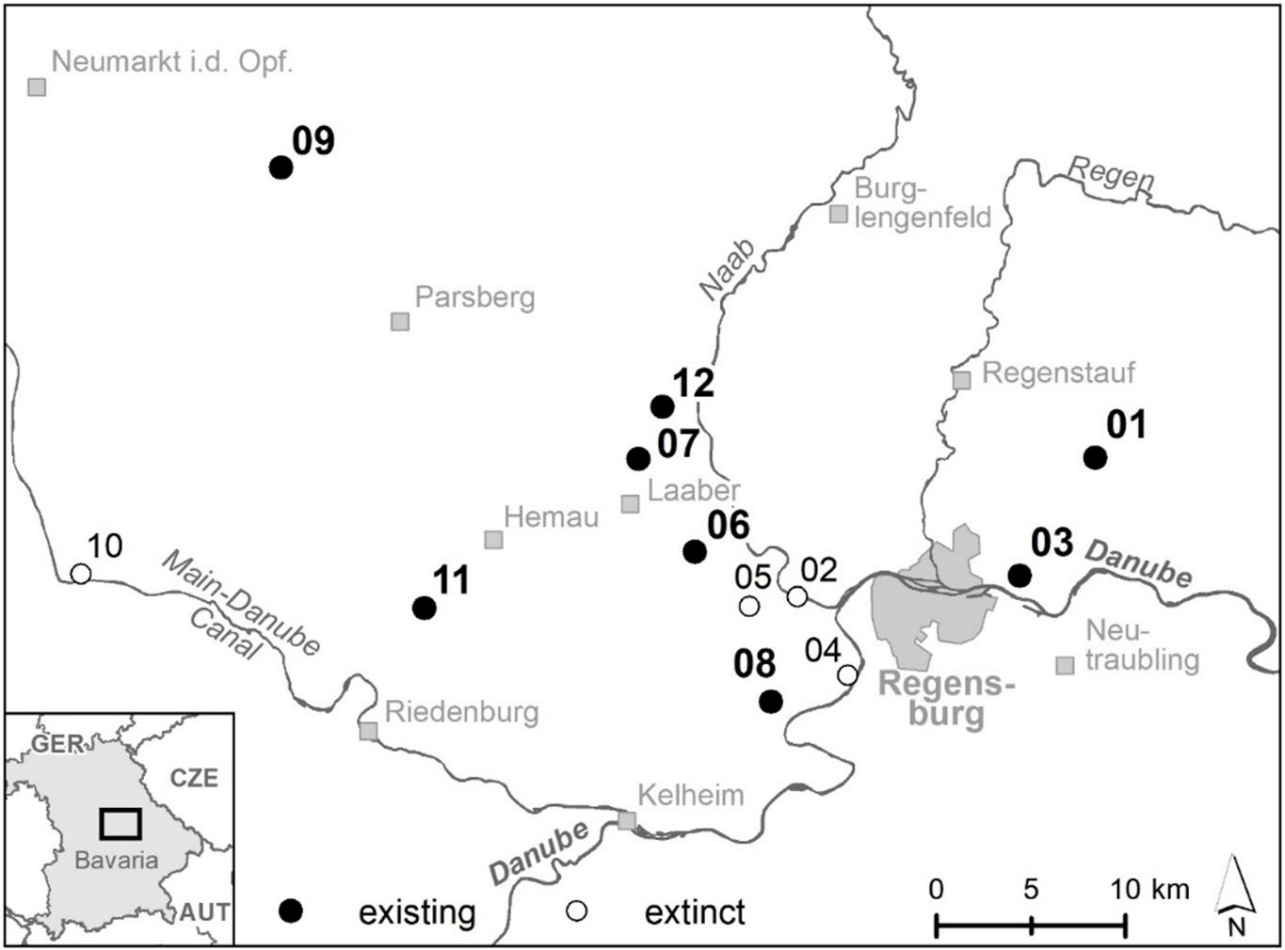
Geographic location of the study sites sampled in 2007 and 2020 around the city of Regensburg (federal state of Bavaria, Germany). Study sites are indicated by black dots. The numbers refer to Listl and Reisch 2014. Sites where *Sherardia arvensis* could not be resampled in 2020, since the populations went extinct, were labelled with white dots.

Based upon the data from a previous study of Listl and Reisch^39^, we selected 12 populations of *S. arvensis* at study sites near Regensburg in southeastern Germany for our investigation (Table 1; Fig. 1), where the species occurred at arable field margins. We revisited all the sites in 2020 to resample *S. arvensis*. However, only at eight study sites *S. arvensis* still occurred. Comparable to the sampling procedure in 2007, at each study site leaf material from ten individuals was collected in 2020, placed in paper filter bags and stored over silica gel to dry at room temperature until DNA extraction in the lab. The plant specimens were identified by two of the current authors in the two sampling years, in 2007 by Daniela Listl and in 2020 by Ellen Gradl respectively. No voucher specimen was collected.

**Table 1:** Studied sites and populations with population ID (Pop ID), location name, status (+: existing, -: extinct) and their geographic location.

Pop ID	Location name	Status	La. (*N*)	Lo. (E)
01	Wenzenbach	+	49°04’51”	12°13’15”
02	Etterzhausen	-	49°01’15”	12°00’04”
03	Tegernheim	+	49°01’35”	12°09’46”
04	Sinzing	-	48°58’57”	12°02’08”
05	Nittendorf	-	49°01’01”	11°57’58”
06	Pollenried	+	49°02’38”	11°55’42”
07	Laaber	+	49°05’20”	11°53’24”
08	Bergmatting	+	48°58’17”	11°58’45”
09	Velburg	+	49°14’01”	11°38’20”
10	Beilngries	-	49°02’38”	11°29’01”
11	Aichkirchen	+	49°01’19”	11°43’52”
12	Heitzenhofen	+	49°06’48”	11°54’33”

### MIG-seq analysis and SNP detection

From the dried plant material collected in 2020, DNA was extracted using the CTAB protocol by Rogers and Bendich^44^, modified by^45^. DNA extracts of the individuals sampled in 2007 had previously been extracted with the same method and had been stored since then at -20 °C in the Regensburg DNA bank. DNA extracts from 2007 to 2020 were diluted with water to a standardized concentration of 15 ng/µL and stored at -20 °C until further analysis. Standardized DNA concentrations are not a requirement for the MIG-seq analysis; however, we would argue that homogenization across samples will lead to more equal numbers of reads per individual and thus more reliable results from the sequencing step.

To analyse the genetic diversity and differentiation of all samples, we conducted multiplexed ISSR (inter simple sequence repeats) genotyping-by-sequencing (MIG-seq) following the protocol by Suyama and Matsuki^42^, which provides genome-wide SNP information. This method is a simple, fast and more cost-efficient approach to obtaining SNPs for ecological questions and non-model species than comparable NGS methods. It has been successfully applied to a variety of study organisms, from corals, snails to different plant species^46–49^ In short, the MIG-seq library was constructed using two PCR steps and fragments between 300 and 800 bp were isolated for sequencing on an Illumina MiSeq Sequencer (Illumina, San Diego, CA, USA) using a MiSeq Reagent Kit v3 (150 cycles, Illumina) as described by Suyama and Matsuki^50^. The raw data was analysed using Trimmomatic, FastQC and Stacks. From the obtained NGS data the primer regions were removed with *Trimmomatic* v.0.36^51^ and the quality check was done with *FastQC* v.0.11.5 (https://www.bioinformatics.babraham.ac.uk/projects/fastqc/). We then analysed the cleaned data with Stacks v.2.66^52^, using the pipeline for de novo SNP discovery which includes the programs *ustacks*,* cstacks*,* sstacks*,* tsv2bam* and *gstacks*. We set the minimum depth coverage at *m* = 3 and the maximum distance at *M* = 2. SNPs were called using the *populations* module in Stacks. To avoid linkages between SNPs we used the *write-single-snp* option, we specified the minimum minor allele frequency (*min-maf* = 0.05), the minimum number of populations which contained retained loci (*p* = 1), maximum observed heterozygosity required to process a locus (*max-obs-het* = 0.8) and retained only those loci that were present in at least 50% of individuals (*min-samples-overall* = 0.5). For comparison we also analysed the dataset with *min-maf* = 0.01 and *min-samples-overall* = 0.6, 0.7 and 0.8 and obtained similar results with regards to the genetic structure. Therefore, only the results with *min-maf* = 0.05 and *min-samples-overall* = 0.5 will be presented here.

### Statistical analysis

From the analysis in stacks using the SNPs, we obtained the mean observed (H_O_) and expected heterozygosity (H_E_) and fixation index within a population (F_IS_) for each population using the populations module.

We used Wilcoxon signed rank tests from the *rstatix* package in R^53^, to check whether study sites differ significantly between 2007 and 2020 in genetic diversity. Furthermore, we analysed genetic differentiation within and among populations, as well as between sampling years using GenAlEx 6.5^54^.

Bayesian cluster analyses were carried out using STRUCTURE^55^ with a burnin and MCMC repetition of 100,000 and 1,000,000 respectively, testing for K = 1–16 with 20 repetitions of each K. We obtained the most likely number of K using the method described by Evanno et al.^56^ with the online tool *Structure Selector*^57^ and R-package *pophelper*^58^. In a second step we ran the structure analysis again on subsets of individuals (Supplementary information 1). The subsets consisted of individuals with more than 50% membership to a primary cluster. To complement the structure analysis, we performed a Discriminant analysis of principal components (DAPC) with the *adegenet*-package^59^. In the first step, in which clusters were detected, we retained all PC axes and choose K when the decrease of BIC reached a plateau (Supplementary information 2). In the second step, that implemented the DAPC, we only retained the first 50 PC axes and retained all discriminant functions.

Additionally, we calculated the effective population size (N_e_) using the software NeEstimator v.2.1^60^. We calculated single-sample contemporary N_e_ using the method based on linkage disequilibrium^61–63^, as well as temporal sample estimates based on Jorde and Ryman’s^64^ estimator as implemented in the software assuming random mating, using 0.05 as the lowest allele frequency threshold and using jackknife-based confidence intervals, similar to the analyses done by Lévêque et al.^65^.

## Results

In total 160 samples were successfully analysed using MIG-seq. After quality filtering, trimming and SNP calling, a total of 371 variable loci were obtained. Mean observed (H_O_) and expected heterozygosity (H_E_) and fixation indices (F_IS_) were nearly identical (Table 2) in 2007 and 2020 (H_O 2007_: 0.17, H_O 2020_: 0.16; H_E 2007_: 0.19, H_E 2020_: 0.20, F_IS 2007_: 0.11, F_IS 2020_: 0.16). Consequently, genetic diversity did not differ significantly between 2007 and 2020 using Wilcoxon tests.

**Table 2:** Genetic variation of the populations of *Sherardia arvensis* at the selected study sites in 2007 and 2020. Given are the population ID (Pop ID), the number of samples included in the analysis N, observed heterozygosity H_O_, expected heterozygosity H_E_ and the fixation index F_IS_. We observed no significant differences between 2007 and 2020 using Wilcoxon tests.

Pop ID	*n*	H_O_	H_E_	F_IS_
2007				
1	10	0.15	0.16	0.06
3	10	0.16	0.2	0.16
6	10	0.17	0.12	-0.07
7	10	0.16	0.24	0.26
8	10	0.17	0.23	0.21
9	10	0.17	0.2	0.15
11	10	0.17	0.22	0.19
12	10	0.16	0.12	-0.05
**Mean 2007**		0.17	0.19	0.11
2020				
1	10	0.17	0.21	0.18
3	10	0.18	0.2	0.12
6	10	0.16	0.21	0.19
7	10	0.16	0.25	0.3
8	10	0.15	0.18	0.14
9	10	0.16	0.21	0.21
11	10	0.16	0.22	0.21
12	10	0.16	0.13	-0.04
**Mean 2020**		0.16	0.2	0.16
***P*** **Wilcoxon test**		**0.64**	**0.31**	**0.25**

In a three level AMOVA (Table 3) including both data from 2007 to 2020 we observed 1% of the total variation between 2007 and 2020, 18% among the populations and 81% within populations. In separate two level AMOVAs (Table 3) genetic differentiation among populations was only slightly stronger in 2007 (Φ_PT_ = 0.19) than in 2020 (Φ_PT_ = 0.18).

**Table 3:** Molecular variance within and among populations of *Sherardia arvensis* from the study sites in 2007 and 2020 calculated in different analyses of molecular variance (AMOVA). SS indicates the sum of squares, MS the mean squares, % the proportion of genetic variability. Levels of significance are based on 999 iteration steps.

Level of variation	df	SS	MS	%	Φ_ΠΤ_	*p*
In 2007 and 2020						
Among years	1	1110.28	1110.28	1	0.18	0.001
Among populations within years	14	12788.6	913.47	18		
Within populations	144	40515.8	281.36	81		
In 2007						
Among populations	7	6693.9	956.27	19	0.19	0.001
Within populations	72	20856.2	289.67	81		
In 2020						
Among populations	7	6094.65	870.66	18	0.18	0.001
Within populations	72	19659.6	273.05	82		

**Fig. 2:**
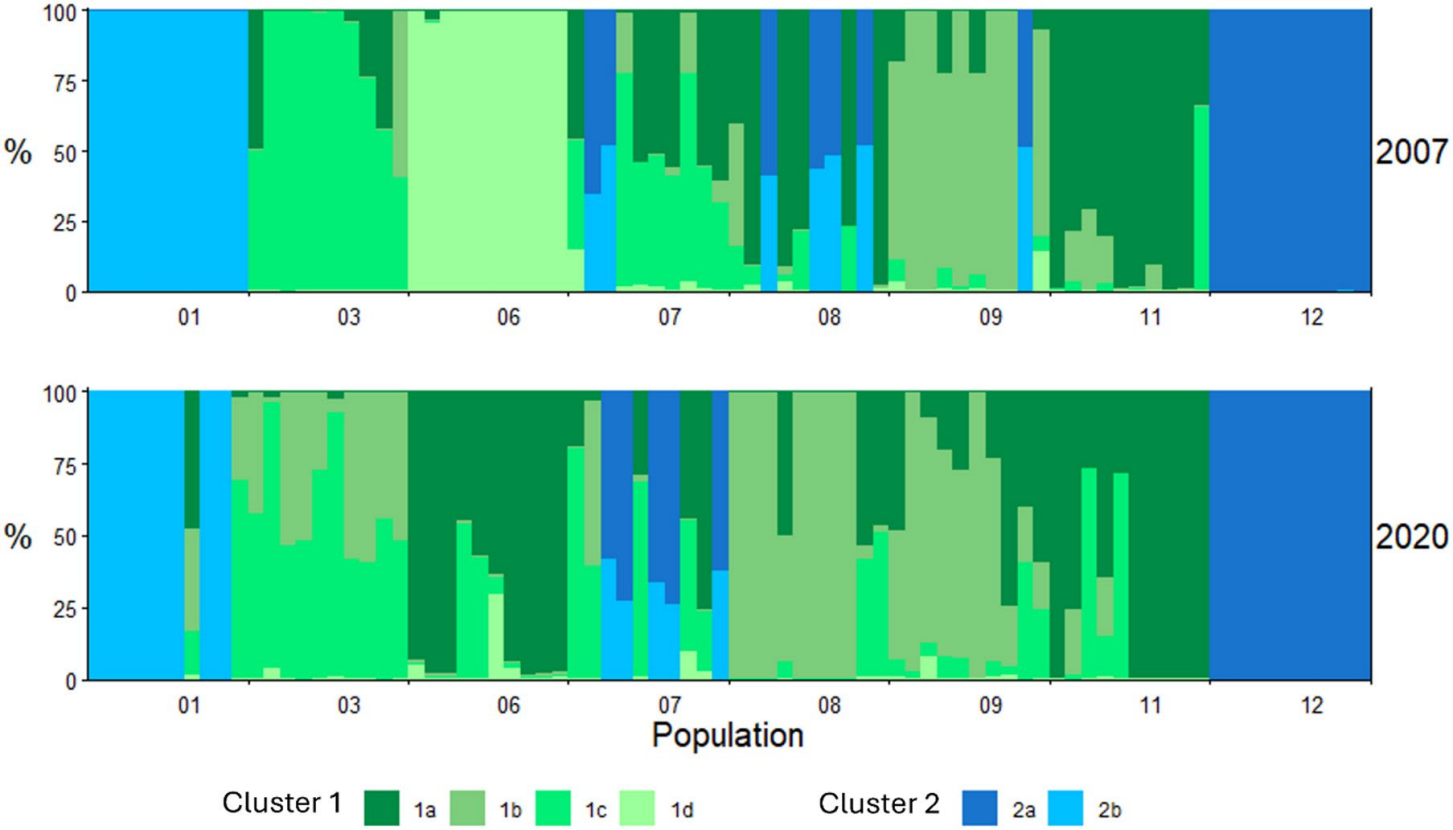
Results of the Bayesian cluster analysis with Structure (based upon SNPs from the MIG-seq analysis) over all sampled individuals simultaneously. Populations from both years of *Sherardia arvensis* were assigned to two main groups (cluster 1 in green and cluster 2 in blue), each of which is sub-structured into several subgroups (1a-1d and 2a and 2b respectively), each bar represents one individual.

**Fig. 3:**
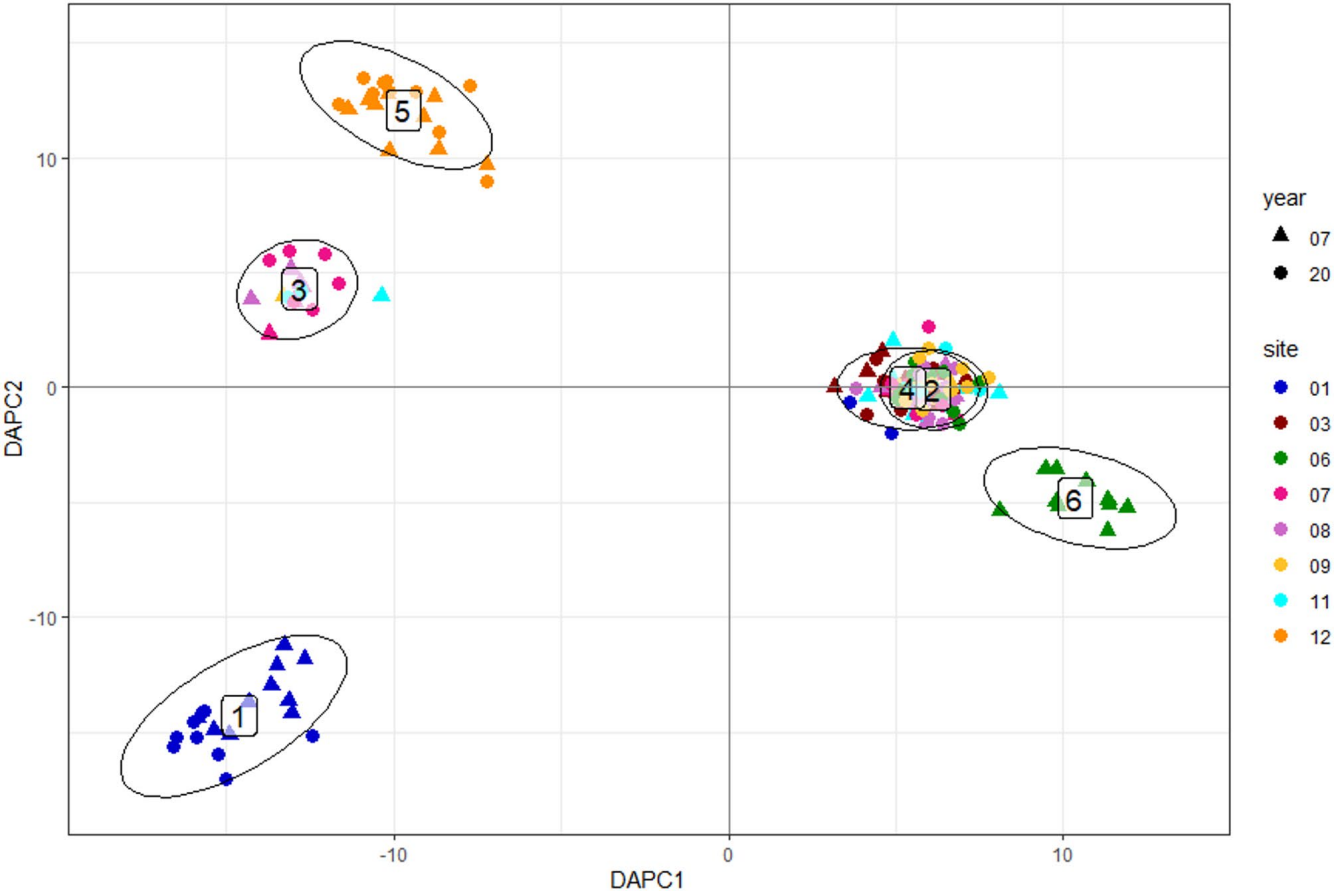
Result of the Discriminant analysis of principal components (DAPC), showing the data structure for six clusters. Samples from 2007 are indicated with a triangle, while samples from 2020 are shown with a dot. The symbols are coloured according to their origin site. Cluster 2 and 4 are overlapping, while clusters 1, 3, 5 and 6 are clearly separated.

In the Bayesian cluster analysis (Fig. 2) individuals and populations formed most likely two main groups (ΔK = 5125.60) for both years. Following the investigation of the lower hierarchy cluster, the first cluster was most likely subdivided into four subclusters, while cluster two was divided into two subclusters (see Supplementary information 1 & 2). These results are further supported by the DAPC analysis (Fig. 3), which shows a similar distribution of samples into the six clusters. Most populations were assigned to the same or similar clusters for both investigated years, while some populations changed more considerably. For example, population 06 was mainly assigned to cluster 1d in 2007 and in 2020 was most likely assigned to cluster 1a, making it more similar to population 11. Similar change patterns were observed for populations 07 and 08. Other populations like population 01, 03, 09, 11 and 12 were more stable between the years.

The estimation of effective population size (Tables 4 and 5) showed different results for the single sample analysis and the temporal method. Single sample estimates changed between the different years, most populations showed a reduction in N_e_, while some showed an increase in N_e_ estimates (population 07 and 09), while population 12 was estimated at infinite for both study years (Table 4). The temporal method showed infinite estimates for most populations, only population 7 (N_e_: 34.03) and population 11 (N_e_: 47.9) showed distinct values, which were however larger than the sample size and the number of individuals present during sampling (personal observation) (Table 5).

**Table 4:** Effective population size estimates for single sample contemporary N_e_ using the method based on linkage disequilibrium (LD) for each single population. Values for the 95% confidence intervals based on a jackknife procedure are represented in square brackets.

		LD method		95% CI
		Sample size	N_e_	
2007	1	10	35.8	[7.5; ∞]
	3	10	56.1	[1.4; ∞]
	6	10	∞	[∞; ∞]
	7	10	15.5	[2.7; ∞]
	8	10	41.5	[3.1; ∞]
	9	10	15.2	[2.5; ∞]
	11	10	11	[2.1; ∞]
	12	10	∞	[∞; ∞]
2020	1	10	5.4	[1.6; ∞]
	3	10	9.1	[1.4; ∞]
	6	10	11.4	[2.4; ∞]
	7	10	∞	[12.1; ∞]
	8	10	2.8	[1.1; ∞]
	9	10	113.9	[3.1; ∞]
	11	10	2.4	[0.9; ∞]
	12	10	∞	[∞; ∞]

**Table 5:** Effective population size estimates for Temporal sample estimates using Jorde & Ryman’s^64^ estimator for each population pair. Values for the 95% confidence intervals based on a jackknife procedure are represented in square brackets.

	Temporal method	*N*_e_	95% CI
	Sample size		
1	20	∞	[∞; ∞]
3	20	∞	[∞; ∞]
6	20	∞	[∞; ∞]
7	20	34.03	[26.3;48.1]
8	20	∞	[∞; ∞]
9	20	∞	[∞; ∞]
11	20	47.9	[34.2;80.3]
12	20	∞	[∞; ∞]

## Discussion

### Loss of populations

For the genetic monitoring study presented here, we revisited 12 study sites, where *S. arvensis* had been sampled in 2007. However, populations of *S. arvensis* still occurred at only 75% of the sites. Our investigation illustrates, therefore, the continuous decline of arable weed populations, which has been previously observed for many species in Central Europe^32,33^.

In the case of *S. arvensis* agricultural land use change may be a key reason for the observed population decline. Fields where *S. arvensis* went seemingly extinct, are meanwhile used for the cultivation of maize instead of cereals. Since arable weeds are light-demanding species and compete with crop plants for light as the main resource^66^ the shadier environmental conditions at maize field margins may have caused the population loss.

Another reason for the population decline may be the changing climate conditions in central Europe. Previous studies showed that many rare arable weed species require low germination temperatures^67^. Furthermore, populations seem to be specifically adapted in seed germination to temperature conditions at their growing sites^68^. Moreover, vegetation strongly suffered from droughts in the recent decade^69^. It has been demonstrated that especially the germination response of rare arable weeds is sensitive to reduced water availability^70^. The increased temperatures and lack of water during the recent decade may, therefore, have contributed further to the observed loss of *S. arvensis* populations.

### Trends in genetic diversity and differentiation patterns

The level of genetic diversity was within the range to be expected for short lived, selfing plant species, which is generally lower than in longer lived and outbreeding plant species^71,72^. The investigation of effective population size showed declining numbers for some of the populations in the single-sample estimates, while others increased. The temporal approached showed infinite or larger values than the census size, similar to Lévêque et al.^65^, who also found different values depending on the method used to estimate N_e_. Unfortunately, most single sample estimates, if not infinite, were below the currently recommended threshold of N_e_ of 50, which is often considered to be the baseline to preserve genetic diversity in wild populations^16,17,27^. However, estimates of Ne are also expected to be lower in species of conservation concern, than in non-threatened species^17^.

In contrast to our expectations, the genetic monitoring we conducted here revealed similar levels of genetic diversity and differentiation within and among the studied populations in 2007 and 2020. At first glance, this seems to be inconsistent when considering the ongoing loss of populations and the preceding decline of population size in arable weeds in general, which should be associated closely with significant changes in the genetic structure, especially in the form of reduced genetic diversity, increasing effects of inbreeding and increasing genetic differentiation.

There is, however, one important reason for the observed more or less stable pattern of genetic diversity and differentiation, which is the longevity of *S. arvensis* seeds in the soil seed bank. Arable weed species often have long-living seeds as revealed by a previous study on arable weeds, where 50% of the analysed species persisted more than 20 years in the soil seed bank. In the same study a soil seed bank longevity of up to 10 years has been reported for *S. arvensis*^42^. The availability of seeds in the soil, may thus buffer populations against extinction and the loss of genetic variation^73–76^. Previous studies in other plant species revealed similar levels of genetic diversity and differentiation in the present vegetation and the soil seed bank. The soil seed bank may thus be considered as a source of hidden diversity and part of the effective population size^76,77^ and thus a buffer against loss of genetic diversity^76^, which could have contributed significantly to the current preservation of genetic diversity and differentiation levels in *S. arvensis* as well as counteracting possible effects of inbreeding, also under conditions of ongoing population decline and increasing isolation.

Our study hence supports the assumption that the reaction of plant populations to decreasing population size and increasing isolation may be delayed, which is often postulated when patterns of genetic diversity and differentiation do not match present patterns of fragmentation^78^. Recent studies already demonstrated that actually existing levels of genetic diversity and differentiation within and among populations of specific species often reflect historical patterns of distribution^78,79^ comparable to an extinction debt at the species level^80,81^. Obviously, longer periods of time are needed to analyse temporal changes of genetic diversity and differentiation. The results of our study show that, at present, *S. arvensis* is more strongly endangered due to the ongoing loss of populations than to a loss of genetic diversity and increase of genetic differentiation and inbreeding. However, the decrease in effective population size in many of the studied populations in the single sample estimates might be an indication, that the buffering capability of the soil seed bank is declining. Similarly, the infinite values obtained in the temporal approach could be a product of the relatively small sample size and actually represent an overestimation of available genetic diversity in the populations^16,17^. But, some populations also increased in their estimates of effective population size between the two study years. It is possible that this may be caused by current or historic gene flow among populations, which are geographically close and might be part of a larger metapopulation, which could explain the shifts in genetic diversity and differentiation found in this study. Historic and present land-use has been repeatedly found to influence current genetic variation patterns in grassland habitats^79,82,83^ and might thus also be of concern in arable weed populations, which experience similar human impacts.

Our investigation, therefore, clearly underlines the need for long-term genetic monitoring over several decades, accompanied by demographic monitoring of a larger sample size and experimental studies to reveal current and future human impacts on genetic diversity and differentiation in the Anthropocene.

## Supplementary Information

Below is the link to the electronic supplementary material.

Supplementary Material 1

Supplementary Material 2

## Data Availability

The datasets generated during the current study are available in the European Nucleotide Archive (ENA) under the accession number PRJEB85125.

## References

[1] Johnson, C. N. et al. Biodiversity losses and conservation responses in the anthropocene. *Science* **356**, 270–275 (2017). 28428393 10.1126/science.aam9317

[2] Eichenberg, D. et al. Widespread decline in central European plant diversity across six decades. *Glob Change Biol.* **27**, 1097–1110 (2021). 10.1111/gcb.15447 33326165

[3] Kruess, A. & Tscharntke, T. Habitat fragmentation, species loss, and biological control. *Science* **264**, 1581–1584 (1994). 17769603 10.1126/science.264.5165.1581

[4] Exposito-Alonso, M. et al. Genetic diversity loss in the anthropocene. *Science* **377**, 1431–1435 (2022). 36137047 10.1126/science.abn5642

[5] Almeida-Rocha, J. M. et al. The impact of anthropogenic disturbances on the genetic diversity of terrestrial species: A global meta‐analysis. *Mol. Ecol.* **29**, 4812–4822 (2020). 33058295 10.1111/mec.15688

[6] Margules, C. R. *Conservation Biology: The Science of Scarcity and Diversity*, JSTOR (1987).

[7] Frankham, R. et al. *Introduction to conservation genetics*, Cambridge university press (2002).

[8] Juergens, N. Monitoring of biodiversity. *Biodivers. Struct. Funct. -I*. **1**, 229 (2009).

[9] Kempel, A. et al. Nationwide revisitation reveals thousands of local extinctions across the ranges of 713 threatened and rare plant species. *Conserv. Lett.* **13**, e12749 (2020).

[10] Lüttgert, L. et al. Repeated habitat mapping data reveal gains and losses of plant species. *Ecosphere* **13**, e4244 (2022).

[11] Kull, T. et al. Necessity and reality of monitoring threatened European vascular plants. *Biodivers. Conserv.* **17**, 3383–3402 (2008).

[12] Straubinger, C. et al. Effects of historical management on the vegetation and habitat properties of wet meadows in Germany. *Restor. Ecol.* **31**, e13839 (2023).

[13] Pearman, P. B. et al. Monitoring of species’ genetic diversity in Europe varies greatly and overlooks potential climate change impacts. *Nat. Ecol. Evol.* **8**, 267–281 (2024). 38225425 10.1038/s41559-023-02260-0 PMC10857941

[14] CBD. Decision Adopted by the Conference of the Parties to the Convention on Biological Diversity CBD/COP/DEC/15/4 Kunming-Montreal Global Biodiversity Framework (2022).

[15] Hoban, S. et al. Global genetic diversity status and trends: towards a suite of essential biodiversity variables (EBVs) for genetic composition. *Biol. Rev.* **97**, 1511–1538 (2022). 35415952 10.1111/brv.12852 PMC9545166

[16] Waples, R. S. The idiot’s guide to effective population size. *Mol. Ecol.*10.1111/mec.17670 (2025). 39925199 10.1111/mec.17670

[17] Clarke, S. H. et al. Global assessment of effective population sizes: consistent taxonomic differences in meeting the 50/500 rule. *Mol. Ecol.* **33**, e17353 (2024). 38613250 10.1111/mec.17353

[18] Thormann, I. et al. Genotypic and phenotypic changes in wild barley (Hordeum vulgare subsp. spontaneum) during a period of climate change in Jordan. *Genet. Resour. Crop Evol.* **64**, 1295–1312 (2017).

[19] Bonnin, I. et al. Explaining the decrease in the genetic diversity of wheat in France over the 20th century. *Agric. Ecosyst. Environ.* **195**, 183–192 (2014).

[20] Khoury, C. K. et al. Crop genetic erosion: Understanding and responding to loss of crop diversity. *New. Phytol*. **233**, 84–118 (2022). 34515358 10.1111/nph.17733

[21] Forgiarini, C. et al. The impact of ex situ cultivation on the genetic variation of endangered plant species – Implications for restoration. *Biol. Conserv.* **284**, 110221 (2023).

[22] Lauterbach, D. et al. Rapid genetic differentiation between ex situ and their in situ source populations: an example of the endangered Silene Otites (Caryophyllaceae). *Bot. J. Linn. Soc.* **168**, 64–75 (2012).

[23] Brütting, C. et al. Ex situ cultivation affects genetic structure and diversity in arable plants. *Plant. Biol.* **15**, 505–513 (2013). 22882447 10.1111/j.1438-8677.2012.00655.x

[24] Morris, A. B. et al. Genetic variation and structure in natural and reintroduced populations of the endangered legume, pyne’s ground Plum (Astragalus bibullatus). *Conserv. Genet.* **22**, 443–454 (2021).

[25] Wei, X. & Jiang, M. Meta-analysis of genetic representativeness of plant populations under ex situ conservation in contrast to wild source populations. *Conserv. Biol.* **35**, 12–23 (2021). 10.1111/cobi.13617 32840007

[26] Finger, A. et al. Genetic monitoring for effective plant conservation: an example using the threatened *Saxifraga hirculus* L. in Scotland. *PLANTS PEOPLE PLANET.* **6**, 381–398 (2024).

[27] Hoban, S. et al. Genetic diversity targets and indicators in the CBD post-2020 global biodiversity framework must be improved. *Biol. Conserv.* **248**, 108654 (2020).

[28] Hoban, S. et al. Global Commitments to Conserving and Monitoring Genetic Diversity Are Now Necessary and Feasible. *BioScience* 71, 964–976 (2021). 10.1093/biosci/biab054 PMC8407967 34475806

[29] Hunter, M. E. et al. Next-generation conservation genetics and biodiversity monitoring. *Evol. Appl.* **11**, 1029–1034 (2018). 30026795 10.1111/eva.12661 PMC6050179

[30] Korneck, D. et al. Warum Verarmt unsere flora? Auswertung der Roten liste der farn-und Blütenpflanzen Deutschlands. *Schriftenreihe Für Veg.* **29**, 299–444 (1998).

[31] Lang, M. et al. Reintroduction of rare arable plants in extensively managed fields: effects of crop type, sowing density and soil tillage. *Agric. Ecosyst. Environ.* **306**, 107187 (2021).

[32] Meyer, S. et al. Dramatic losses of specialist arable plants in central Germany since the 1950s/60s – a cross-regional analysis. *Divers. Distrib.* **19**, 1175–1187 (2013).

[33] Richner, N. et al. Dramatic decline in the Swiss arable flora since the 1920s. *Agric. Ecosyst. Environ.* **241**, 179–192 (2017).

[34] Schneider, C. et al. *Biologisch-ökologische Grundlagen des Schutzes gefährdeter Segetalpflanzen*, Bundesamt für Naturschutz (1994).

[35] Fried, G. et al. Arable weed decline in Northern france: crop edges as refugia for weed conservation? *Biol. Conserv.* **142**, 238–243 (2009).

[36] Brooker, R. W. et al. Facilitation and sustainable agriculture: a mechanistic approach to reconciling crop production and conservation. *Funct. Ecol.* **30**, 98–107 (2016).

[37] Metzing, D. et al. Rote Liste gefährdeter Tiere, Pflanzen und Pilze Deutschlands. Band 7: Pflanzen. In 7 (2018).

[38] Scheuerer, M. & Ahlmer, W. Rote liste gefährdeter Gefäßpflanzen Bayerns Mit regionalisierter florenliste. *Schriftenreihe Bayer Landesamtes Für Umweltschutz*. **165**, 1–372 (2003).

[39] Listl, D. & Reisch, C. Genetic variation of Sherardia arvensis L. – How land use and fragmentation affect an arable weed. *Biochem. Syst. Ecol.* **55**, 164–169 (2014).

[40] Jäger, E. J. et al. (eds) *Exkursionsflora Von Deutschland* (Spektrum Akademischer, 2013).

[41] Knuth, P. *Handbuch der Blütenbiologie*, Engelmann (1898).

[42] Wäldchen, J. et al. Zur Diasporen-Keimfähigkeit von Segetalpflanzen. *Beitr. Für Forstwirtsch Landschaftsökologie*. **39**, 145–156 (2005).

[43] Poschlod, P. *Geschichte Der Kulturlandschaft: Entstehungsursachen Und Steuerungsfaktoren Der Entwicklung Der Kulturlandschaft, Lebensraum- Und Artenvielfalt in Mitteleuropa* 2nd edn (Verlag Eugen Ulmer, 2017).

[44] Rogers, S. O. & Bendich, A. J. Extraction of total cellular DNA from plants, algae and fungi. In Plant Molecular Biology Manual (eds, S. B. &, R. A.) 183–190 (Springer Netherlands, 1994).

[45] Reisch, C. Genetic structure of Saxifraga tridactylites (Saxifragaceae) from natural and man-made habitats. *Conserv. Genet.* **8**, 893–902 (2007).

[46] Kusuma, Y. W. C. et al. Genetic diversity and structure of *Hopea bilitonensis*, an endemic dipterocarp from Belitung Island, Indonesia. *J. Asia-Pac Biodivers.* **17**, 400–405 (2024).

[47] Takata K, Taninaka H, Nonaka M, Iwase F, Kikuchi T, Suyama Y, Nagai S, Yasuda N. 2019. Multiplexed ISSR genotyping by sequencing distinguishes two precious coral species (Anthozoa: Octocorallia: Coralliidae) that share a mitochondrial haplotype. PeerJ 7:e7769, 10.7717/peerj.7769 10.7717/peerj.7769 PMC6779117 31598424

[48] Hirano, T. et al. Role of ancient lakes in genetic and phenotypic diversification of freshwater snails. *Mol. Ecol.* **28**, 5032–5051 (2019). 31617614 10.1111/mec.15272

[49] Sakaba, T. et al. Phylogeography of the temperate grassland plant tephroseris Kirilowii (Asteraceae) inferred from multiplexed inter-simple sequence repeat genotyping by sequencing (MIG-seq) data. *J. Plant. Res.* **136**, 437–452 (2023). 37148377 10.1007/s10265-023-01452-w

[50] Suyama, Y. & Matsuki, Y. MIG-seq: an effective PCR-based method for genome-wide single-nucleotide polymorphism genotyping using the next-generation sequencing platform. *Sci. Rep.* **5**, 16963 (2015). 26593239 10.1038/srep16963 PMC4655332

[51] Bolger, A. M. et al. Trimmomatic: a flexible trimmer for illumina sequence data. *Bioinformatics* **30**, 2114–2120 (2014). 24695404 10.1093/bioinformatics/btu170 PMC4103590

[52] Catchen, J. et al. Stacks: an analysis tool set for population genomics. *Mol. Ecol.* **22**, 3124–3140 (2013). 23701397 10.1111/mec.12354 PMC3936987

[53] Kassambara, A. rstatix: Pipe-Friendly Framework for Basic Statistical Tests 0.7.2 (2019).

[54] Peakall, R. & Smouse, P. E. GenAlEx 6.5: genetic analysis in Excel. Population genetic software for teaching and research—an update. *Bioinformatics* **28**, 2537–2539 (2012). 22820204 10.1093/bioinformatics/bts460 PMC3463245

[55] Pritchard, J. K. et al. Documentation for structure software: Version 2.3.

[56] Evanno, G. et al. Detecting the number of clusters of individuals using the software structure: a simulation study. *Mol. Ecol.* **14**, 2611–2620 (2005). 15969739 10.1111/j.1365-294X.2005.02553.x

[57] Li, Y. & Liu, J. StructureSelector: A web-based software to select and visualize the optimal number of clusters using multiple methods. *Mol. Ecol. Resour.* **18**, 176–177 (2018). 28921901 10.1111/1755-0998.12719

[58] Francis, R. M. Pophelper: an R package and web app to analyse and visualize population structure. *Mol. Ecol. Resour.* **17**, 27–32 (2017). 26850166 10.1111/1755-0998.12509

[59] Jombart, T. & Ahmed, I. *Adegenet 1.3-1*: new tools for the analysis of genome-wide SNP data. *Bioinformatics* **27**, 3070–3071 (2011). 21926124 10.1093/bioinformatics/btr521 PMC3198581

[60] Do, C. et al. NeEstimator v2: re-implementation of software for the estimation of contemporary effective population size (*N*_*e*_) from genetic data. *Mol. Ecol. Resour.* **14**, 209–214 (2014). 23992227 10.1111/1755-0998.12157

[61] Waples, R. S. A bias correction for estimates of effective population size based on linkage disequilibrium at unlinked gene loci*. *Conserv. Genet.* **7**, 167–184 (2006).

[62] Waples, R. S. & Do, C. Linkage disequilibrium estimates of contemporary *N*_e_ using highly variable genetic markers: a largely untapped resource for applied conservation and evolution. *Evol. Appl.* **3**, 244–262 (2010). 25567922 10.1111/j.1752-4571.2009.00104.x PMC3352464

[63] Hill, W. G. Estimation of effective population size from data on linkage disequilibrium. *Genet. Res.* **38**, 209–216 (1981).

[64] Jorde, P. E. & Ryman, N. Unbiased estimator for genetic drift and effective population size. *Genetics* **177**, 927–935 (2007). 17720927 10.1534/genetics.107.075481 PMC2034655

[65] Lévêque, A. et al. Levels and Spatial patterns of effective population sizes in the Southern damselfly (*Coenagrion mercuriale*): on the need to carefully interpret Single-Point and Temporal estimations to set conservation guidelines. *Evol. Appl.* **17**, e70062 (2024). 39720624 10.1111/eva.70062 PMC11667679

[66] Colbach, N. et al. The response of weed and crop species to shading: which parameters explain weed impacts on crop production? *Field Crops Res.* **238**, 45–55 (2019).

[67] Rühl, A. T. et al. Future challenge for endangered arable weed species facing global warming: low temperature Optima and narrow moisture requirements. *Biol. Conserv.* **182**, 262–269 (2015).

[68] Bürger, J. et al. Populations of arable weed species show intra-specific variability in germination base temperature but not in early growth rate. *PLOS ONE*. **15**, e0240538 (2020). 33035273 10.1371/journal.pone.0240538 PMC7546504

[69] Reinermann, S. et al. The effect of droughts on vegetation condition in germany: an analysis based on two decades of satellite Earth observation time series and crop yield statistics. *Remote Sens.* **11**, 1783 (2019).

[70] Rühl, A. T. et al. Distinct germination response of endangered and common arable weeds to reduced water potential. *Plant. Biol.* **18**, 83–90 (2016). 25786499 10.1111/plb.12331

[71] Reisch, C. & Bernhardt-Römermann, M. The impact of study design and life history traits on genetic variation of plants determined with AFLPs. *Plant. Ecol.* **215**, 1493–1511 (2014).

[72] Duminil, J. et al. Plant traits correlated with generation time directly affect inbreeding depression and mating system and indirectly genetic structure. *BMC Evol. Biol.* **9**, 177 (2009). 19635127 10.1186/1471-2148-9-177 PMC2728730

[73] Yang, X. et al. Global patterns of potential future plant diversity hidden in soil seed banks. *Nat Commun* **12**, 7023 (2021). 10.1038/s41467-021-27379-1 10.1038/s41467-021-27379-1 PMC8639999 34857747

[74] Ottewell, K. M. et al. Can a seed bank provide demographic and genetic rescue in a declining population of the endangered shrub acacia pinguifolia? *Conserv. Genet.* **12**, 669–678 (2011).

[75] Honnay, O. et al. Can a seed bank maintain the genetic variation in the above ground plant population? *Oikos* **117**, 1–5 (2008).

[76] Vitalis, R. et al. When genes go to sleep: the population genetic consequences of seed dormancy and monocarpic perenniality. *Am. Nat.* **163**, 295–311 (2004). 14970929 10.1086/381041

[77] Iberl, K. et al. A source of hidden diversity: soil seed bank and aboveground populations of a common herb contain similar levels of genetic variation. *Plant. Biol.* **25**, 1035–1045 (2023). 37703520 10.1111/plb.13571

[78] Münzbergová, Z. et al. Historical habitat connectivity affects current genetic structure in a grassland species. *Plant. Biol.* **15**, 195–202 (2013). 22646655 10.1111/j.1438-8677.2012.00601.x

[79] Reisch, C. et al. Genetic diversity of calcareous grassland plant species depends on historical landscape configuration. *BMC Ecol.* **17**, 19 (2017). 28438203 10.1186/s12898-017-0129-9 PMC5404287

[80] Krauss, J. et al. Habitat fragmentation causes immediate and time-delayed biodiversity loss at different trophic levels. *Ecol. Lett.* **13**, 597–605 (2010). 20337698 10.1111/j.1461-0248.2010.01457.x PMC2871172

[81] Helm, A. et al. Slow response of plant species richness to habitat loss and fragmentation. *Ecol. Lett.* **9**, 72–77 (2006). 16958870 10.1111/j.1461-0248.2005.00841.x

[82] Lehmair, T. A. et al. Genetic variation of litter meadow species reflects gene flow by hay transfer and mowing with agricultural machines. *Conserv. Genet.* **21**, 879–890 (2020).

[83] Reisch, C. et al. Drivers of genetic diversity in plant populations differ between semi-natural grassland types. *Biodivers. Conserv.* **30**, 3549–3561 (2021).

