# Supplementary material for: Genetic monitoring of an endangered arable weed reveals local maintenance of genetic variation in times of land use and climate change
Source: Sci Rep. 2026 Feb 4;16:4991. doi: 10.1038/s41598-026-38363-4 (PMC12877195; doi:10.1038/s41598-026-38363-4)

**Supplementary information 2**

Supplementary Figure S1: Output of the DAPC analysis, the curve starts to plateau at 6 clusters.
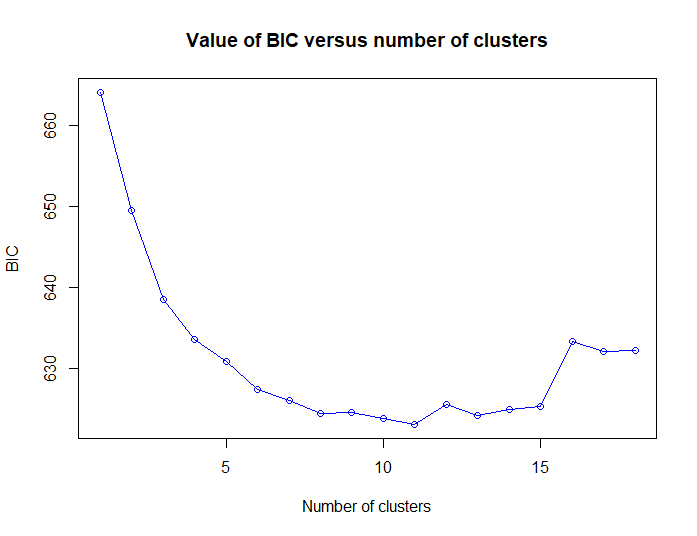

Supplement: Supplementary file 1 — Supplementary Material 1 [file 41598_2026_38363_MOESM1_ESM.docx]
