# Supplementary material for: Genetic monitoring of an endangered arable weed reveals local maintenance of genetic variation in times of land use and climate change
Source: Sci Rep. 2026 Feb 4;16:4991. doi: 10.1038/s41598-026-38363-4 (PMC12877195; doi:10.1038/s41598-026-38363-4)
Supplement: Supplementary file 2 — Supplementary Material 2 [file 41598_2026_38363_MOESM2_ESM.pdf]

Bayesian Cluster analysis Sherardia arvensis

Explore K 1-18

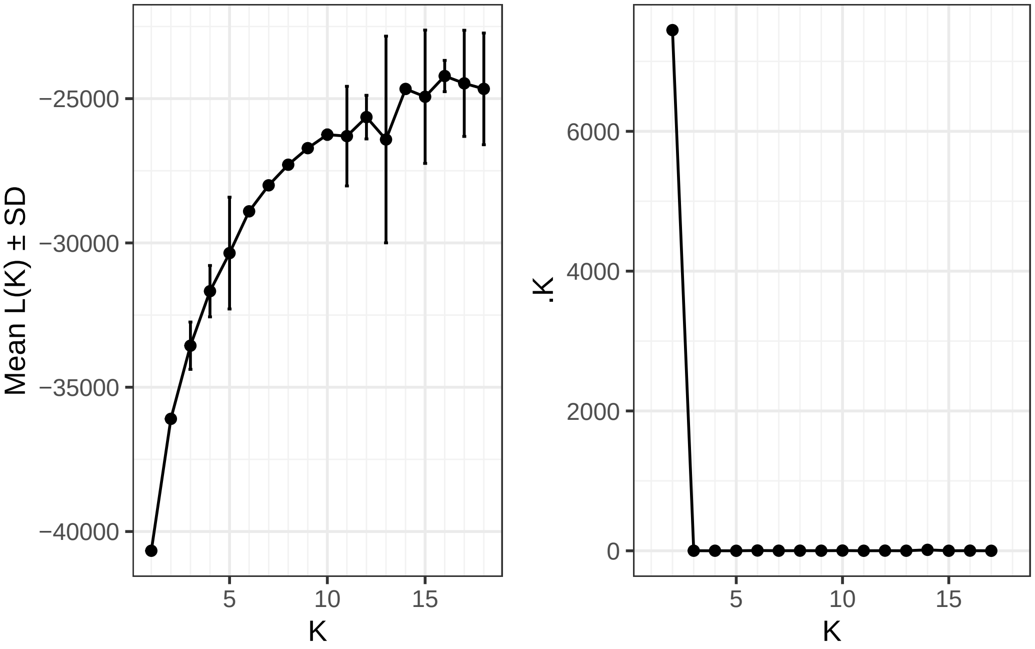

Delta(K) peaks at K= 2, L(K) has no clear peak.

Clumpak K = 2

detected mode: 20/20

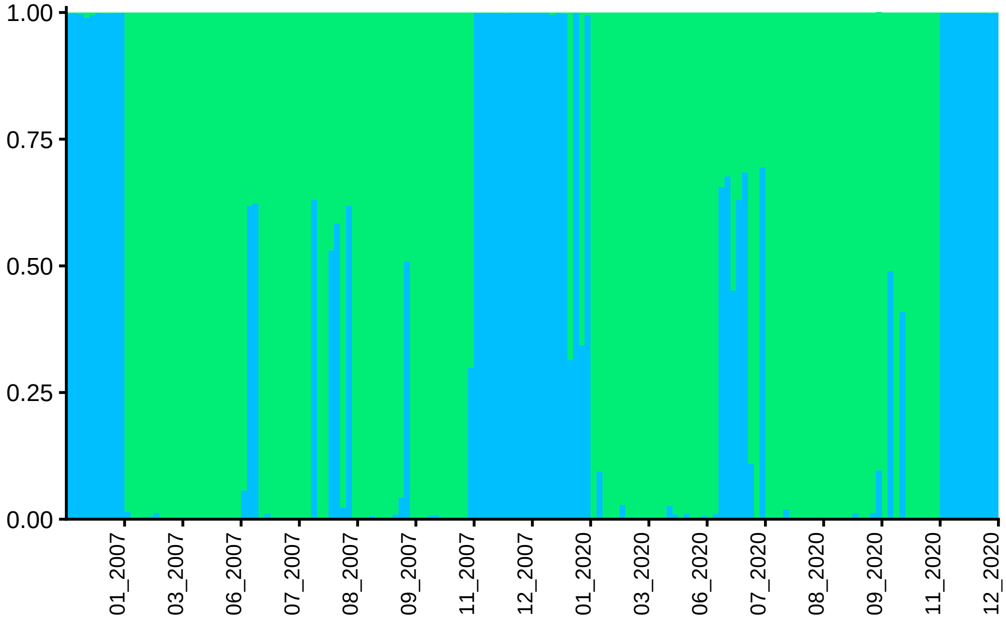

Investigate lower hierarchy

Cluster 1

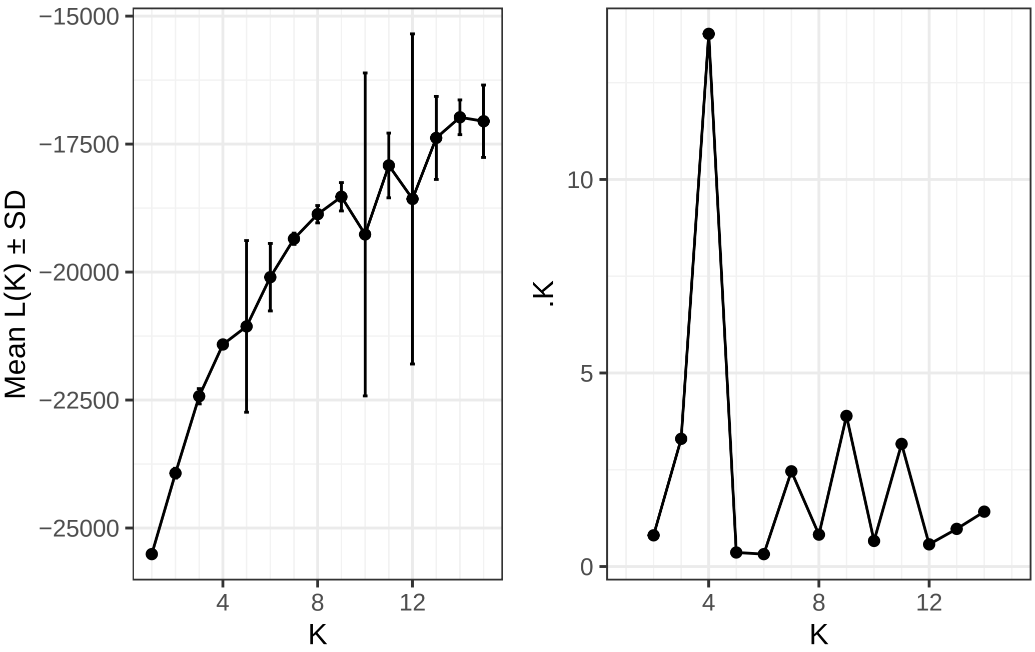

delta(K) peaks at K=4, L(K) has no clear peak, but first plateau after K=4.

Clumpak K = 4

Detected mode: 14/20

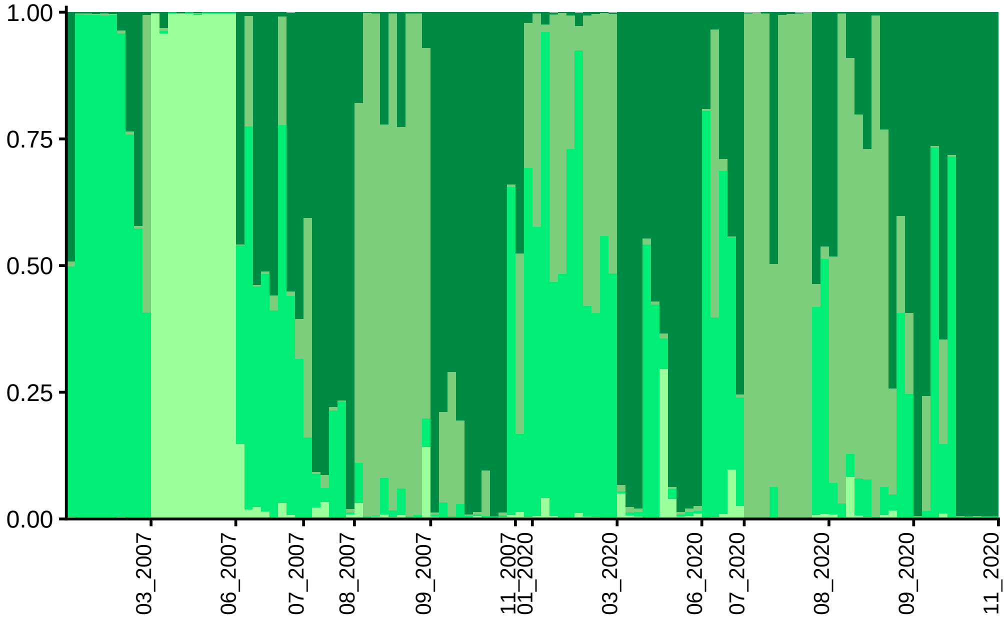

detected mode: 6/20

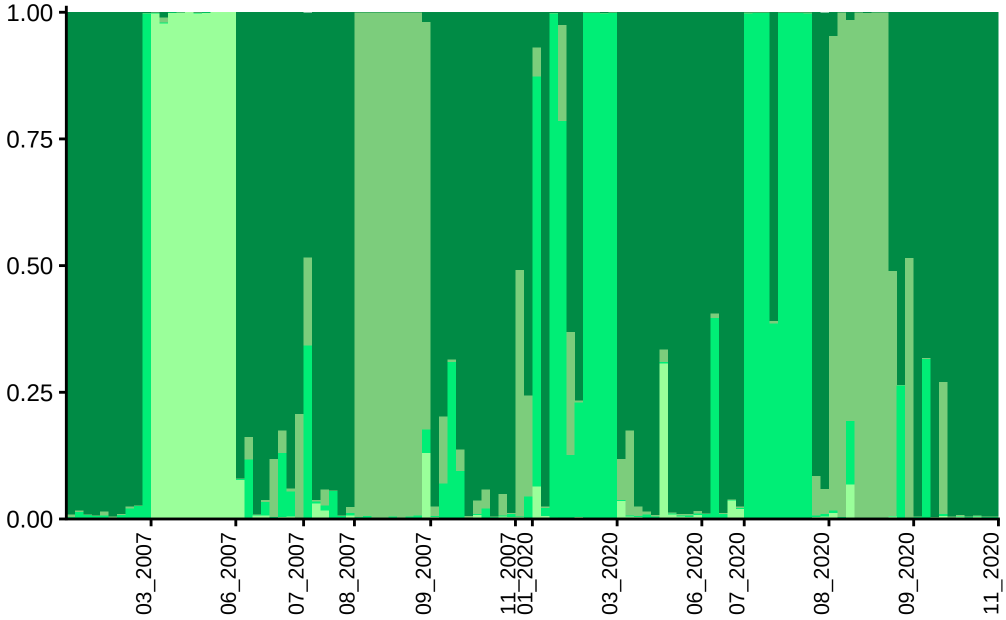

Cluster 2

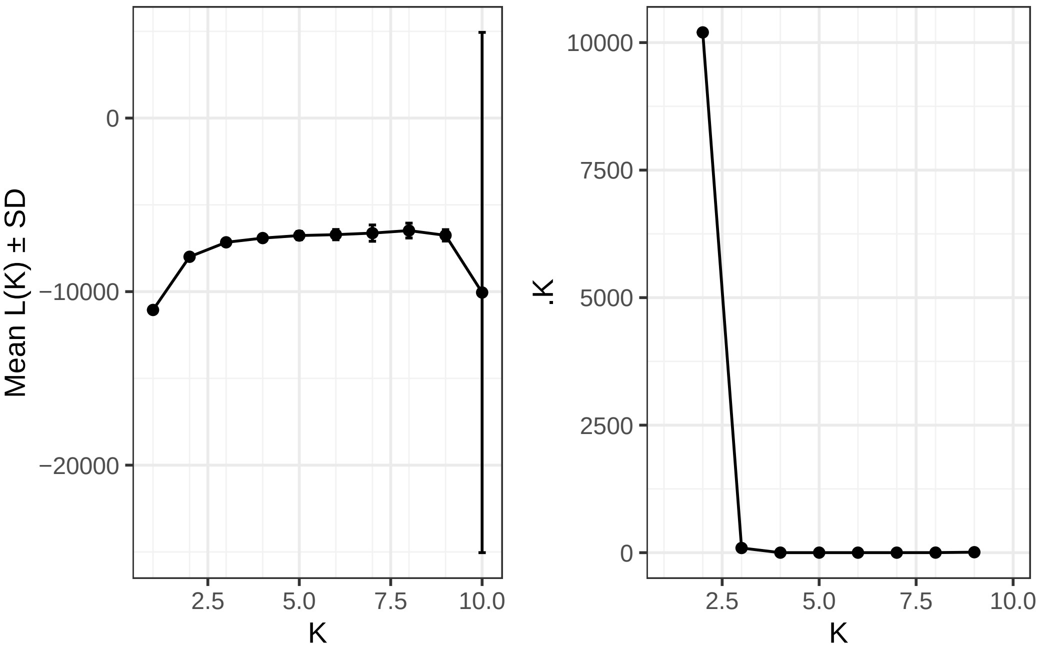

delta(K) peaks at K = 2, L(K): plateau begins at K = 2

Clumpak K=2

detected mode: 20/20

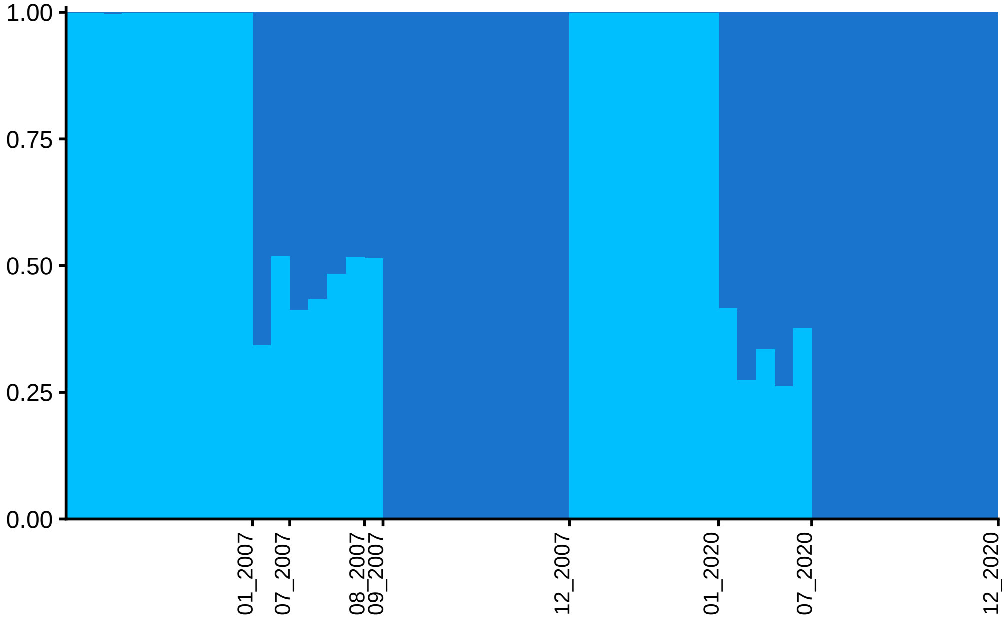

Combined subclusters

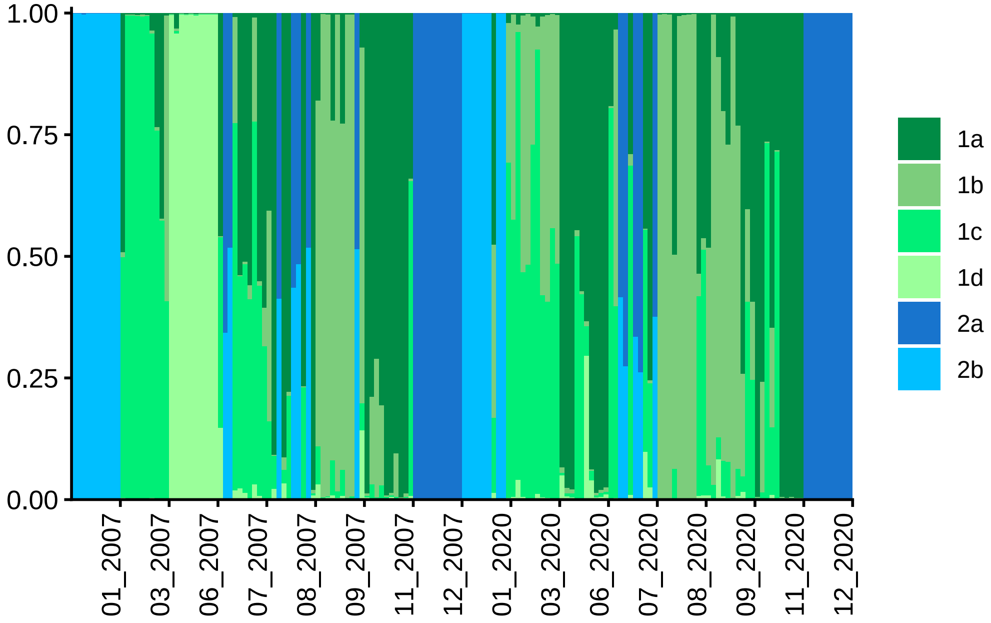

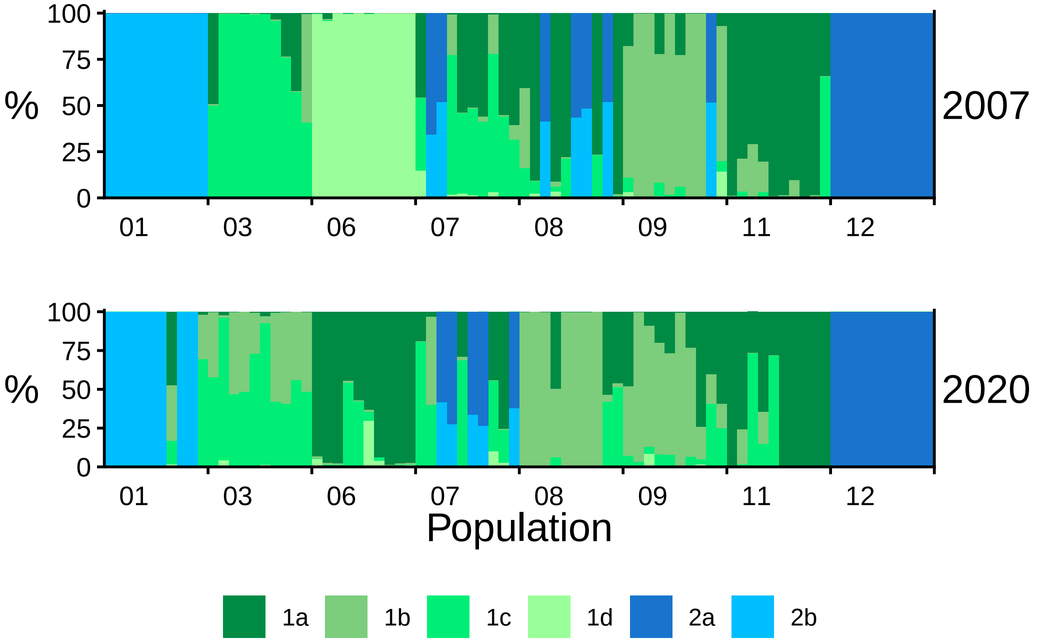
100
75
50
25
0
%
2007
01
03
06
07
08
09
11
12
100
75
50
25
0
%
2020
01
03
06
07
08
09
11
12
Population
1a
1b
1c
1d
2a
2b
